# Safety of Sublingual Immunotherapy: A Secondary Analysis of Post-marketing Adverse Events Reports Using a Japanese Public Database

**DOI:** 10.7759/cureus.45177

**Published:** 2023-09-13

**Authors:** Yudai Kaneda, Uiri Kaneda, Mira Namba, Tetsuya Tanimoto

**Affiliations:** 1 School of Medicine, Hokkaido University, Sapporo, JPN; 2 Faculty of Foreign Languages, Dokkyo University, Soka, JPN; 3 School of Medicine, Keio University, Tokyo, JPN; 4 Internal Medicine, Accessible Rail Medical Services Tetsuikai, Navitas Clinic Tachikawa, Tokyo, JPN

**Keywords:** sublingual immunotherapy, allergic rhinitis, miticure®, cedarcure®, actair®, allergic rhinitis (ar), sublingual immunotherapy (slit)

## Abstract

Purpose

Allergic rhinitis impacts a significant portion of the Japanese population, leading to the rise of sublingual immunotherapy (SLIT) as an alternative treatment. Despite its growing popularity, there is limited safety information. Therefore, this study aimed to consolidate data on its adverse effects in an academic context.

Methods

We conducted a secondary analysis of adverse events reported in the Pharmaceutical Adverse Events Information Database for three SLIT drugs (Actair®, Cedarcure®, and Miticure®) approved in Japan. A descriptive analysis concerning age, gender, underlying diseases, symptoms, time of onset, and outcomes was performed.

Results

We identified 98 cases of adverse reactions reported for the SLIT drugs. These cases were mainly from the pediatric to adolescent group (73.7%). Males made up 59.5% of reports. Recovery or improvement was noted in 97.7% of reports. Anaphylactic reactions were the most common adverse event (42.6%), followed by respiratory distress (12.2%). Reactions typically occurred within one week of starting treatment (54.1%).

Conclusions

Our research illuminated the safety of SLIT drugs in Japan, revealing a favorable profile. It underscores the need for vigilance, particularly among younger patients and during initial doses, emphasizing the importance of proper patient selection and further research to enhance the treatment's efficacy.

## Introduction

Allergic rhinitis (AR) is reported to have a prevalence exceeding 40% in Japan, with an estimated patient population surpassing 30 million, making it one of the most common ailments [1]. The proportion of patients has been estimated to increase by approximately 10 percentage points every decade, and it is associated with not only rhinitis but also troublesome symptoms occasionally akin to asthma that may compromise the quality of life, productive time at work or school, sleep quality, and participation in outdoor activities [2]. Standard medical therapy consists of pharmacological treatment, generally the use of non-sedating oral antihistamines, topical nasal antihistamines, and intranasal corticosteroid sprays, in conjunction with allergen avoidance when possible [2]. While these medications are effective, they do not impact the underlying allergic predisposition, necessitating long-term use if symptoms recur [3]. For AR patients whose symptoms remain controlled inadequately despite conventional therapies, consideration of allergen immunotherapy is warranted [3].

In Japan, sublingual immunotherapy (SLIT) has received increasing attention recently. This treatment involves the daily administration of small quantities of allergenic substances throughout three to five years, gradually acclimating the body to these allergens [4]. SLIT is considered safer than injecting allergenic substances, and the possibility of self-administration at home enhances patient convenience. Allergen immunotherapy has been recommended for all severities and types of perennial AR and hay fever in guidelines such as the Allergic Disease Diagnosis and Treatment Guidelines 2020 and the Nasal Allergy Treatment Guidelines 2020 and has been approved for national insurance coverage since 2014 in Japan [5-7]. Firstly, SLIT for Japanese cedar pollen allergy among adults was granted market approval in 2014, followed by SLIT for dust mite allergy in 2015, thus initiating therapies against the two major allergens responsible for AR in the country [5].

Furthermore, since 2018, treatment has been available for children aged five and above for cedar pollen and dust mite allergies. In Japan, as of 2023, three SLIT drugs that are available for use include a 300 IR SLIT tablet (Actair®, Stallergenes Greer, Antony, France; Shionogi & Co., Ltd., Osaka, Japan), house dust mite sublingual tablet (Miticure®, Torii Pharmaceutical, Tokyo, Japan), and Japanese cedar pollen sublingual tablet (Cedarcure®, Torii Pharmaceutical). For dust mite allergies, studies have shown favorable effects in children and adults with the use of Miticure® [8]. Additionally, it has been found that treatment with Miticure® for one year results in efficacy for approximately 80% of individuals, with nasal symptoms reduced by about 50% [9].

However, SLIT is relatively new, with limited numbers of safety data in the real world, and post-marketing data on the adverse reactions associated with SLIT are limited. As the government discloses adverse event reports for marketed drugs in a publicly available database in Japan, we conducted a secondary analysis of safety data concerning SLIT to summarize the characteristics of adverse events after marketing approval.

## Materials and methods

Actair®

Actair® is a pharmaceutical product manufactured by Shionogi & Co., Ltd. and is used for desensitization therapy against AR caused by mite antigens. It is typically administered once daily via sublingual administration. The initial dose is 100 units, with subsequent increases of 100 units every three days, up to a maximum of 300 units. The main adverse effects listed in the package insert include urticaria, pruritus, erythema and skin redness, stomach pain, nausea, vomiting, and diarrhea.

Cedarcure®

Cedarcure® is a desensitization therapeutic drug for Japanese cedar pollen allergy, sold by Torii Pharmaceutical Co., Ltd. It is marketed as a cedar pollen sublingual tablet 2000JAU. For the first week after starting the medication, one tablet of the product (containing 2,000JAU as the main ingredient) is held under the tongue for one minute once daily, then swallowed. From the second week onward, a daily dose of 5000JAU is taken. The main adverse effects listed in the package insert include stomatitis, sublingual swelling, pharyngeal pruritus, and headache.

Miticure®

Miticure® is a desensitization therapeutic drug for AR caused by mite antigens, manufactured by Torii Pharmaceutical Co., Ltd. Initially, one tablet of 3,300JAU is administered once daily for one week, followed by a daily dose of one 10,000JAU tablet from the second week onward. The main adverse effects listed in the package insert include oral edema, pruritus, and pharyngeal irritation.

Publicly available pharmaceutical adverse events database

Pharmaceutical Adverse Events Information Database [10], provided by the Pharmaceuticals and Medical Devices Agency (PMDA) in Japan, compiles reports regarding suspected adverse events of pharmaceuticals and medical devices reported within the country. It is made publicly available to serve as a reference material for healthcare professionals and researchers. The information contained in the database is compiled from various sources, including spontaneous adverse event reports from healthcare practitioners and patients, reports based on the PMDA's post-marketing surveillance survey forms, which are conducted to collect safety information after a product has been marketed, and information gathered from hearings with pharmaceutical companies.

Methodology

Our study systematically searched the Pharmaceutical Adverse Events Information Database to comprehensively extract adverse event information associated with SLIT drugs approved in Japan. We aimed to capture all adverse event reports related to the approved SLIT drugs in Japan from the beginning of their sales up to July 17, 2023. Notably, the liquid formulation, Cedartolen®, was first introduced to the market in 2014; however, its sales were discontinued around 2019 and subsequently replaced by the tablet form, Cedarcure® [11]. As a result, adverse event reports related to Cedartolen® were excluded from the search in this study.

Utilizing the in-built search functionalities of the database, we individually inputted the names of the three SLIT drugs: Actair®, Cedarcure®, and Miticure®. This ensured a focused retrieval of relevant cases associated with each medication, and all the reported cases identified were included. Throughout the search process, a descriptive analysis was performed concerning age, gender, underlying diseases, symptoms of adverse events, the time of onset, and outcomes. However, when specific reporting items were labeled as "Unknown," such data were not included in the denominator for percentage calculations to ensure accuracy and relevance.

Ethical approval

Ethical considerations were not applied to this study, as only publicly available data were used.

## Results

**Figure 1:**
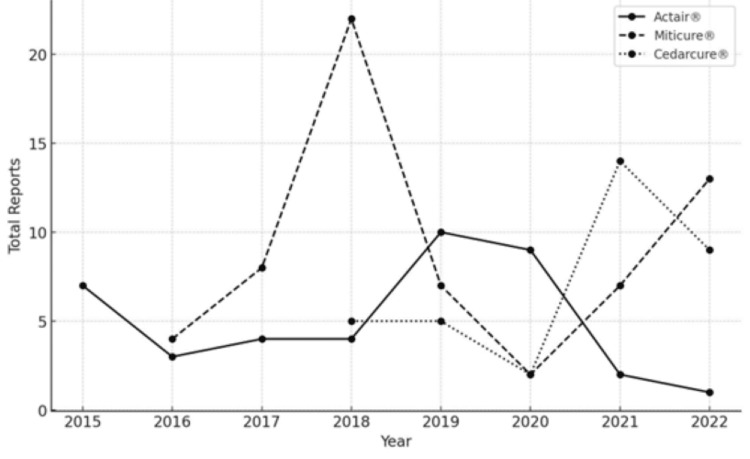
Total reports per year for each medication

A total of 98 cases of SLIT were reported in the pharmaceutical adverse events database, with 31 cases for Actair®, 24 cases for Cedarcure®, and 41 cases for Miticure®. Figure 1 shows the number of reported cases for each fiscal year. The highest number was 22 cases for Cedarcure® in 2018, and the lowest was one case for Actair® in 2022, but there were at least five cases in total every year since the start of sales in 2015.

Table 1 shows the background information of patients who reported adverse reactions to each medication. Overall, the reports were predominantly from the pediatric to adolescent group, with 73.7% (70/95) from individuals aged under 20 years. Males accounted for 59.5% (56/94) of the cases. The outcome was recovery or improvement in 97.7% (86/88) of the reports. The two cases reported as unrecovered were IgA nephropathy in a teenage female using Actair® and eosinophilic gastroenteritis in a female under 10 years old using Miticure®. Reports involving only one underlying disease (such as AR, hypersensitivity, or seasonal allergies) constituted 37.8% (34/90), with the majority having multiple underlying diseases; AR was found in 83.3% (75/90), seasonal allergies were found in 31.1% (28/90), asthma was found in 18.9% (17/90), and atopic dermatitis in 12.2% (11/90).

**Table 1:** The background information of patients who reported adverse reactions to each medication

	Actair® (N = 31)	Cedarcure® (N = 24)	Miticure® (N = 43)	Total (N = 98)
Age				
Child (age unspecified)	2 (6.5%)	2 (8.3%)	2 (4.7%)	4 (4.1%)
0-10	5 (16.1%)	4 (16.7%)	9 (20.9%)	18 (18.4%)
10-19	14 (45.2%)	8 (33.3%)	26 (60.5%)	48 (49.0%)
20-29	1 (3.2%)	0	1 (2.3%)	2 (2.0%)
30-39	3 (9.7%)	3 (12.5%)	0	6 (6.1%)
40-49	3 (9.7%)	2 (8.3%)	4 (9.3%)	9 (9.2%)
50-59	2 (6.5%)	1 (4.2%)	1 (2.3%)	4 (4.1%)
60-69	1 (3.2%)	3 (12.5%)	0	4 (4.1%)
Unknown	0	2 (8.3%)	1 (2.3%)	3 (3.1%)
Sex				
Male	19 (61.3%)	9 (37.5%)	28 (65.1%)	56 (57.1%)
Female	10 (32.3%)	14 (58.3%)	14 (32.6%)	38 (38.8%)
Unknown	2 (6.5%)	1 (4.2%)	1 (2.3%)	4 (4.1%)
Major primary illness				
Allergic rhinitis	27 (90.0%)	9 (45.0%)	39 (97.5%)	75 (83.3%)
Seasonal allergies	2 (6.7%)	18 (90.0%)	8 (20.0%)	28 (31.1%)
Asthma	4 (13.3%)	6 (30.0%)	7 (17.5%)	17 (18.9%)
Atopic dermatitis	6 (20.0%)	2 (10.0%)	3 (7.5%)	11 (12.2%)
Unknown	1	4	3	8

Table 2 shows information on the outcomes of each medication. The most common adverse events were anaphylactic reactions at 42.6% (42/98), respiratory distress at 12.2% (12/98), and asthma at 7.1% (7/98). Occurrences within one week of the initial administration were 54.1% (46/85), and during dose increases were 4.7% (4/85). Upon re-administration, adverse events were reported in one out of eight cases (12.5%).

**Table 2:** Adverse effects caused by each medication and their outcomes

	Actair® (N = 31)	Cedarcure® (N = 24)	Miticure® (N = 43)	Total (N = 98)
Major adverse effects				
Anaphylactic reactions	11 (35.5%)	9 (37.5%)	22 (51.2%)	42 (42.9%)
Respiratory distress	2 (6.5%)	3 (12.5%)	7 (16.3%)	12 (12.2%)
Asthma	2 (6.5%)	2 (8.3%)	3 (7.0%)	7 (7.1%)
Timing of adverse effects				
<1 week	20 (69.0%)	11 (57.9%)	15 (40.5%)	46 (54.1%)
During dose increases	3 (10.3%)	1 (5.3%)	0	4 (4.7%)
Other	6 (20.7%)	7 (36.8%)	22 (59.5%)	35 (41.2%)
Unknown	2	5	6	13
Outcomes				
Recovery	25 (83.3%)	16 (80.0%)	30 (78.9%)	71 (80.7%)
Mild recovery	4 (13.3%)	4 (20.0%)	7 (18.4%)	15 (17.0%)
Unrecovered	1 (3.3%)	0	1 (2.6%)	2 (2.3%)
Unknown	1	4	5	10

For Actair®, 31 adverse effects were reported, with five cases (16.1%) in children under 10 years, 14 cases (45.2%) in teenagers, and two cases (6.5%) in children of indeterminate age. Remarkably, 21 cases (67.7%) were reported among individuals under 20 years. The gender distribution comprised 19 males (61.2%) and 10 females (32.3%), with two cases (6.5%) remaining unidentified. Outcomes were characterized by 25 recoveries (80.6%), four mild improvements (12.9%), one non-recovery (3.2%), and one unknown (3.2%). The most frequently reported side effect was anaphylaxis, with 11 cases (35.5%), followed by laryngeal edema, asthma, and respiratory distress, each with two cases (6.5%). A case that did not recover was reported to have IgA nephropathy.

Cedarcure® had 24 reported adverse effects, with four cases (16.7%) under 10 years, eight cases (33.3%) in teenagers, and one case (4.2%) in children of unknown age, making a total of 13 cases (54.2%) from individuals under 20 years. Among these, there were nine males (37.5%), 14 females (58.3%), and one unknown (4.2%). Outcomes included 16 recoveries (66.7%), four mild improvements (16.7%), zero non-recoveries, and four unknown (16.7%). Adverse effects were predominantly anaphylaxis with nine cases (37.5%), followed by respiratory distress with three cases (12.5%), and other effects such as allergic gastroenteritis and toxic rash reported once each (4.2%).

Miticure® had 43 reported adverse effects, with nine cases (20.9%) under 10 years, 26 cases (60.5%) in teenagers, and one case (2.3%) in children of unknown age, resulting in 36 cases (83.7%) from individuals under 20 years. The gender distribution was 28 males (65.1%), 14 females (32.6%), and one unknown (2.3%). Outcomes encompassed 30 recoveries (69.8%), seven mild improvements (16.3%), one non-recovery (2.3%), and five unknown (11.6%). The most frequent adverse effect was an anaphylactic reaction with 22 cases (51.1%), followed by breathing difficulty with seven cases (16.3%), voice disorder with four cases (9.3%), and laryngeal edema and asthma with three cases (7.0%) each. Other symptoms included pharyngeal swelling, chest discomfort, and low blood pressure. Eosinophilic gastroenteritis was reported in the case that did not recover.

## Discussion

Our study offers an analysis of adverse events reported by three marketed SLIT drugs (Actair®, Cedarcure®, and Miticure®) available in Japan as of 2023. During the designated observation timeframe after market approval, 98 adverse effects were identified and distributed among the drugs as follows: Actair® (31), Cedarcure® (24), and Miticure® (41). These incidents predominantly occurred in individuals younger than 20 years, often alongside multiple underlying health conditions. Despite these findings, nearly all cases were deemed recovered or improved, and also, in re-administration cases, adverse effect reports were observed only in one out of eight cases, affirming the treatments' relative safety.

The side effects ranged from anaphylactic reactions to respiratory issues. One plausible reason for these adverse reactions, especially anaphylactic reactions, may be due to patients' hypersensitivity to the antigens present in the SLIT drugs [12]. Furthermore, individual variations in immune response and the presence of underlying health conditions can exacerbate these reactions [13]. To mitigate these risks, it is imperative to enforce rigorous patient oversight, particularly during the treatment's initial week or when dosages are increased. Comprehensive patient education, stringent candidate selection, and meticulous treatment monitoring are also crucial [14]. Patients with specific medical conditions, like labile asthma or those on beta blockers, may not be suitable for SLIT [14], emphasizing the importance of careful candidate selection.

The study's demographic findings, revealing a higher prevalence of adverse effects in males under 20 years, call for further investigation into potential age and gender-related vulnerabilities to SLIT-related reactions. Developing age-specific guidelines may foster more effective, tailored SLIT treatment strategies. Additionally, two unrecovered cases, IgA nephropathy with Actair® and eosinophilic gastroenteritis with Miticure®, urge caution in determining causality, as they manifested during stabilized administration periods.

The Japanese government's intent to encourage SLIT utilization for AR with an ambitious goal to quadruple supply within five years aligns with the Japanese Ministry of Health, Labor, and Welfare's plans to promote allergen immunotherapy [15]. In the context of this government initiative, it is reasonable to forecast a substantial increase in SLIT sales in Japan. However, our findings illuminate potential risks, especially anaphylactic reactions among the youth, necessitating an impartial evaluation of SLIT's efficacy, safety, cost-effectiveness, and patient suitability. Consideration must also be extended to potential domestic preferences influencing drug selection, mirroring the skepticism surrounding domestically produced Shionogi's Xocova® emergency approval during the novel coronavirus disease 2019 (COVID-19) pandemic [16].

Our study's limitations include reliance on publicly accessible databases and possibly omitting mild, unreported reactions or those physicians dismiss. On the other hand, in the PMDA database used in this report, items such as the correlation with adverse effects and the denominator of prescriptions are not disclosed, akin to the COVID-19 vaccine adverse reaction reporting database [17,18]. This absence of information may result in potential biases, such as increased onset frequency or a propensity for reports to be more prevalent in pediatric cases. Therefore, caution is required in the interpretation of these findings. The descriptive nature and absence of causality judgment criteria within the dataset further compound the potential biases.

## Conclusions

This secondary analysis sheds light on the safety profile of SLIT drugs in Japan. The findings indicate a generally favorable safety profile but also highlight the importance of continuous vigilance, proper patient selection, and the need for further research to optimize these promising treatments. As a foundational study, this study beckons more expansive inquiries to refine SLIT's applicability in managing allergic conditions in Japan, thereby contributing positively to patient quality of life.
